# Dual mechanisms of grain refinement in a FeCoCrNi high-entropy alloy processed by high-pressure torsion

**DOI:** 10.1038/srep46720

**Published:** 2017-04-21

**Authors:** Wenqian Wu, Min Song, Song Ni, Jingshi Wang, Yong Liu, Bin Liu, Xiaozhou Liao

**Affiliations:** 1 State Key Laboratory of Powder Metallurgy, Central South University, Changsha 410083, China; 2 School of Aerospace, Mechanical and Mechatronic Engineering, The University of Sydney, Sydney, NSW 2006, Australia

## Abstract

An equiatomic FeCoCrNi high-entropy alloy with a face-centered cubic structure was fabricated by a powder metallurgy route, and then processed by high-pressure torsion. Detailed microscopy investigations revealed that grain refinement from coarse grains to nanocrystalline grains occurred mainly via concurrent nanoband (NB) subdivision and deformation twinning. NB–NB, twin–NB and twin–twin interactions contributed to the deformation process. The twin–twin interactions resulted in severe lattice distortion and accumulation of high densities of dislocations in the interaction areas. With increasing strain, NB subdivision and interactions between primary twins and inclined secondary stacking faults (SFs)/nanotwins occurred. Secondary nanotwins divided the primary twins into many equiaxed parts, leading to further grain refinement. The interactions between secondary SFs/nanotwins associated with the presence of Shockley partials and primary twins also transformed the primary twin boundaries into incoherent high-angle grain boundaries.

High-entropy alloys (HEAs) have received considerable attention, and become one of the most promising structural materials since their discovery in 2004^1^,^2^. HEAs were originally defined as homogenous solid-solution alloys with face- and/or body-centered cubic (FCC and/or BCC) structures, comprising at least five metallic elements with content ranging from 5 to 35 at.%^3^,^4^. Recently, HEAs with hexagonal close-packed (HCP) structure were also discovered^5^, and quaternary medium-entropy alloys were also classified as HEAs^6^. Compared to multi-phase HEAs, single-phase HEAs truly conserve their high configurational entropy, stabilize a homogenous solid-solution, and possess a large lattice distortion^7^,^8^.

Severe plastic deformation (SPD) induced grain refinement processes involve complicated microstructural evolutions, which could significantly affect the mechanical behavior of the materials^9^,^10^. Depending on materials, SPD-induced grain refinement can occur mainly by dislocation activities or by combined deformation twinning and dislocation activities^11^. Extensive dislocation activities, including dislocation multiplication, accumulation, interaction, tangling, and spatial rearrangement, can subdivide a large grain into many smaller ones with the formation of dislocation cells and the evolution of cell boundaries into low-angle and then high-angle grain boundaries (GBs). This type of the grain refinement has often been observed in BCC materials including TaNbHfZrTi HEAs^12^, and FCC materials with high stacking fault energies (SFEs) such as nickel^13^ and aluminum alloys^14^. Deformation twinning leads to twin boundary (TB) subdivision and the subsequent dislocation–TB interactions transform coherent TBs into conventional high-angle GBs. The second type of grain refinement mechanism has been widely reported in FCC materials with low SFEs^11^, including CoCrFeNiMn HEA^15^, Al_0.1_CoCrFeNi HEA^16^, and twinning-induced plasticity (TWIP) steels^17^. It has been reported that increasing the density of TBs effectively increases the strength of materials and retains or improves their ductility simultaneously, because TBs impede dislocation slip and also increase the dislocation storage capability^18^,^19^. Deformation twinning is promoted by low SFE, low temperature, and high strain rate^20^. Recent experimental and theoretical investigations indicated that an equiatomic FeCoCrNi HEA has a low SFE (~20–25 mJ m^−2^)^21^.

The plastic deformation mechanisms of HEAs could be different from that of conventional alloys, since HEAs have highly distorted lattice structures due to the different atomic sizes and chemical bonds of their constituent elements^22^. Recently, the microstructure and thermal stability of a nanocrystalline (NC, grain size < 100 nm) CoCrFeNiMn HEA after high-pressure torsion (HPT) were studied^23^, and the result showed that the microstructure was refined to a grain size of ~50 nm along with an exceptional increase of the strength. Tang *et al*. reported that a nanostructure of Al_0.3_CoCrFeNi alloy processed by HPT processing has a high incremental hardness, and subsequent annealing at appropriate temperatures gave an ordered BCC secondary phase with an additional increase in hardness^24^. Yu *et al*. showed that the plastic deformation mechanisms of an Al_0.1_CoCrFeNi FCC HEA induced by HPT at room temperature include dislocation slip at low strains and twinning at high strains^16^.

FeCoCrNi HEA is a stable single FCC solid solution with high toughness even at cryogenic temperatures^25^,^26^. Recently, He *et al*. reported that the FeCoCrNi HEA is metastable at intermediate temperatures^27^. In order to obtain HEAs with great potential applications, many researchers have designed various new HEAs, such as CoCrFeNiMn^15^,^23^,^26^, CoCrFeNiAl_x_^28^ and CoCrFeNiMo_x_^29^, by adding alloying elements to the base FeCoCrNi HEA. As a base alloy, identifying the microstructural evolution of FeCoCrNi alloy during deformation is essential for HEAs on both scientific and industrial aspects. In this study, we performed a systematic investigation of the microstructural evolution of a FeCoCrNi HEA introduced by HPT. The investigation presents detailed information on the grain refinement mechanisms of the HEA, and provides fundamental basis for the applications of FeCoCrNi based HEAs.

## Results

During the unconfined HPT processing, the equivalent von Mises strain (ε) can be calculated from the following equation^30^:


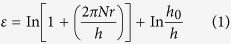


where r is the radius of the disk, N is the number of the revolution, and h_0_ and h are the initial and final thicknesses, respectively. Supplementary Fig. S1 shows the relationship between the hardness and the equivalent strain. The as-received sample had an average hardness of ~218 Hv, and the hardness increased to ~234 Hv after applying a high pressure of 4 GPa for ~1 minute without torsional treatment (N = 0). The hardness increased continuously with increasing strain until it reached a plateau of ~530 Hv. The hardness plateau indicates that the microstructure reached an equilibrium state. Depending on the microstructural characteristics observed in this study and the corresponding equivalent strain, the HPT-induced microstructural evolution process was divided into four deformation stages with increasing strain. The four stages were continuous and overlap to each other because the HPT process was a continuous deformation process. As such, it is difficult to delineate a clear boundary between neighboring deformation stages.

### Deformation stage 1 (ε = ~0–0.25): formation of nanobands (NBs) and deformation twins

**Figure 1:**
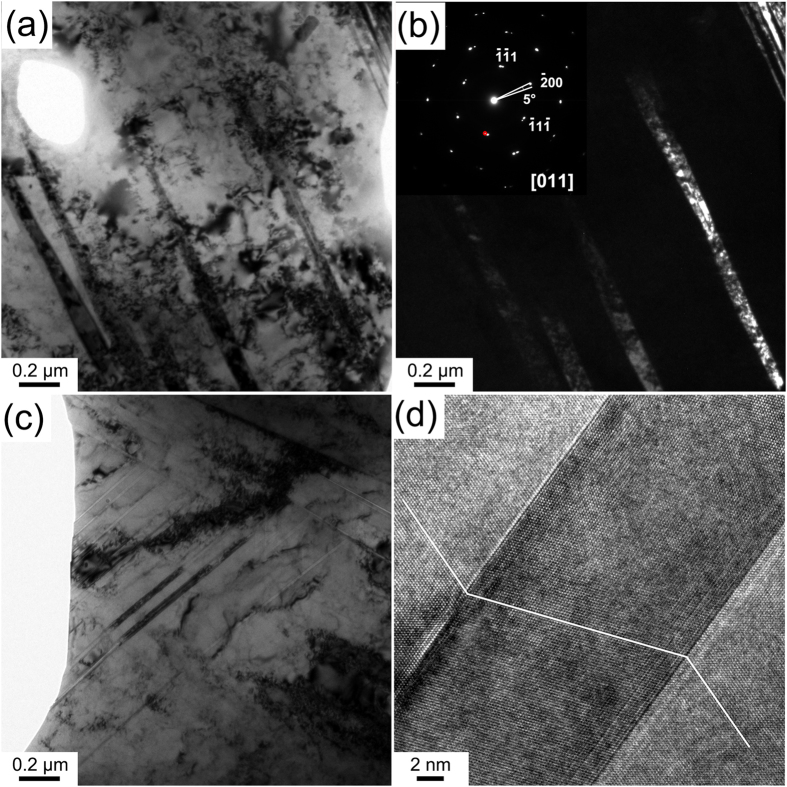
(**a**) A TEM image of a set of NBs; (**b**) its corresponding dark-field image and SAED pattern. The SAED pattern shows misorientation of ~5° between the NBs and the matrix; (**c**) a TEM image of primary deformation twins; and (**d**) an HRTEM image of a primary deformation twin.

In deformation stage 1, grains mainly maintain the original size (~25 μm) and present a microstructure with significant numbers of NBs and deformation twins. Figure 1 illustrates transmission electron microscope (TEM) images in deformation stage 1. A lamellar structure (nanobands) with an average width of ~65 nm is clearly observed in deformation stage 1, as shown in Fig. 1a. Figure 1b shows the corresponding dark-field image of the NBs. The corresponding selected area electron diffraction (SAED) pattern inset in Fig. 1b demonstrates that there is a misorientation angle of ~5° between the NBs and the matrix. The misorientation angle would increase gradually with increasing the deformation strain.

Deformation twins with TB spacing ranging from ~5 nm to ~40 nm were also observed at this deformation stage, as shown in Fig. 1c, in which two active twinning systems were activated. Figure 1d shows a high-resolution TEM (HRTEM) image of a deformation twin. The TBs of deformation twins in this deformation stage were usually coherent and atomic flat with no dislocation blocked at the boundaries.

Although the morphologies of NBs and deformation twins were very similar in this deformation stage, the boundaries of NBs are not as straight as those of the twins. They can also be easily differentiated by electron diffraction along a <110> direction. Further, while TBs were exactly parallel to each other at this deformation stage (see Fig. 1c), NBs in a bundle were only approximately parallel to each other.

### Deformation stage 2 (ε = ~0.25–3.0): NB–NB, twin–NB and twin–twin interactions

**Figure 2:**
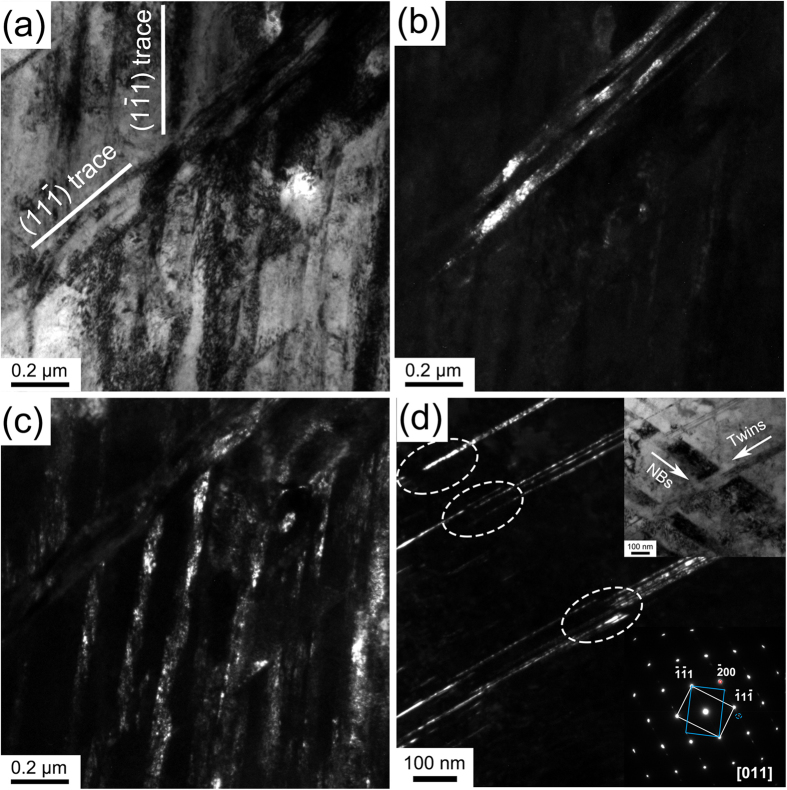
(**a**) A TEM image showing the interaction between two sets of NBs. The two lines mark crystallographic planes of the matrix; (**b**) and (**c**) are the corresponding dark-field images of the two sets of NBs, respectively; (**d**) a dark-field TEM image showing a set of NBs interacting with a set of twins (Note that the interaction regions are marked as ellipses). The insets at the lower right corner and the upper right corner are a corresponding SAED pattern and a bright-field image, respectively.

Three interaction characteristics can be observed in this deformation stage. Figure 2a presents a typical TEM micrograph, showing the interactions between two sets of NBs. The two sets of NBs are parallel to the {111}, which is consistent with previous observations in other alloys^31^,^32^. Figure 2b and c show dark-field images of the two sets of NBs, respectively. It is obvious that the two sets of NBs still remained an unbroken structure, while the boundaries of these complementary sets of the NBs distorted to some extent due to the dislocation generation and accumulation along the boundaries of the NBs with increasing strain.

Figure 2d shows a dark-field image that a group of deformation twins stopped and re-launched after interacting with a set of NBs, in which the interaction areas of the twins are marked by white dashed ellipses. The corresponding SAED pattern (lower right inset in Fig. 2d) indicates a twin relationship. A corresponding bright-field image in upper right inset in Fig. 2d shows that the set of NBs have a width of 80–100 nm, while the interspaces of the individual breaking-up twins in the interaction areas (marked by white dashed ellipses in Fig. 2d) have a width smaller than 80 nm. Tips of these twins were embedded in the NBs at the twin–NB interaction areas. It can be deduced that the NBs acted as obstacles to slip and twinning, in which deformation twins were stopped and re-launched. The formation of these twins inside the area of NBs can be induced by localized shear deformation.

**Figure 3:**
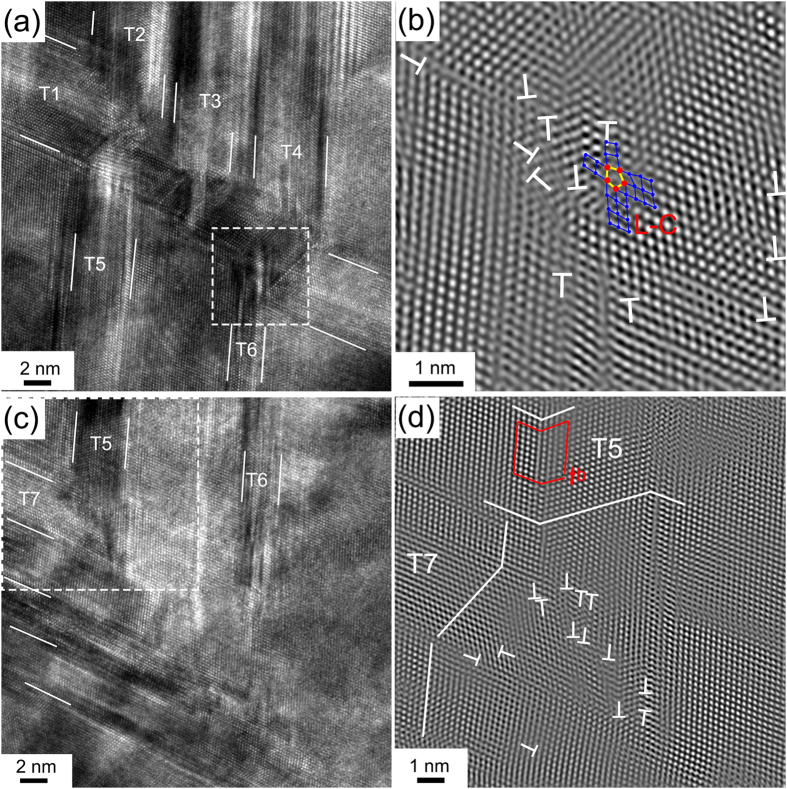
(**a**) An HRTEM image of twin–twin interaction, showing three incident twins (T2, T3 and T4) in the same direction interacting with one barrier twin (T1) and triggering the formation of T5 and T6 twins (Note that the TBs are highlighted by white lines); (**b**) a Fourier filtered image of the framed part in (**a**), showing plenty of dislocations (marked with white “T”) and a L-C lock (indicated by five red spots); (**c**) the T5 twin was stopped by the T7 twin; and (**d**) a Fourier filtered image of the framed part in (**c**), showing plenty of dislocations (marked with white “T”) and a Shockley partial (Burger circuits are outlined by red lines) at TB of T5.

Figure 3 shows twin–twin interactions, which are frequently observed in the deformation stage 2. Figure 3a shows a HRTEM micrograph of twin–twin interactions, in which three close twins (T2, T3 and T4) propagated along the same direction, interacted with the pre-existing twin T1. The interactions led to significant local stress concentration at one side of T1 and the stress was partly released from the other side of T1 through the twinning of T5 and T6. It is interesting to note that T1 was deflected heavily under the total twinning shear of T2, T3 and T4, and became thinner after the interactions. A Fourier filtered image obtained from the twin–twin interaction area marked by a white dashed square in Fig. 3a is shown in Fig. 3b. A high density of dislocations (marked with “T”) and severe lattice distortion were observed near the interacted TBs. Interestingly, a Lomer-Cottrell (L-C) lock was observed in the area, as delineated by the pentagon with five red dots at its corners in Fig. 3b. The L-C lock formed by the reaction of two leading partials from two dissociated 60° lattice dislocations on two intercrossing slip planes, and its structure consists of two stacking faults meeting each other at 70.5° angle and connected by a stair-rod dislocation^33^,^34^. Additionally, the twin (T5) propagated and interacted with a set of pre-existing twins (marked as T7), and was then stopped by the pre-existing T7, as shown in Fig. 3c. Figure 3d shows a Fourier filtered image of the area enclosed by the white square in Fig. 3c, and indicates that the TBs of the T5 are associated with the presence of Shockley partials (as step). Severe lattice distortion occurred and plenty of dislocations (marked with “T”) accumulated in the interaction area in Fig. 3d. Note that the lattice distortion induced by the twin–twin interaction is different from the intrinsic lattice distortion caused by the significant atomic size variation in HEAs.

### Deformation stage 3 (ε = ~3.0–4.0): grain refinement by NBs interaction and formation of secondary stacking faults (SFs)/nanotwins

**Figure 4:**
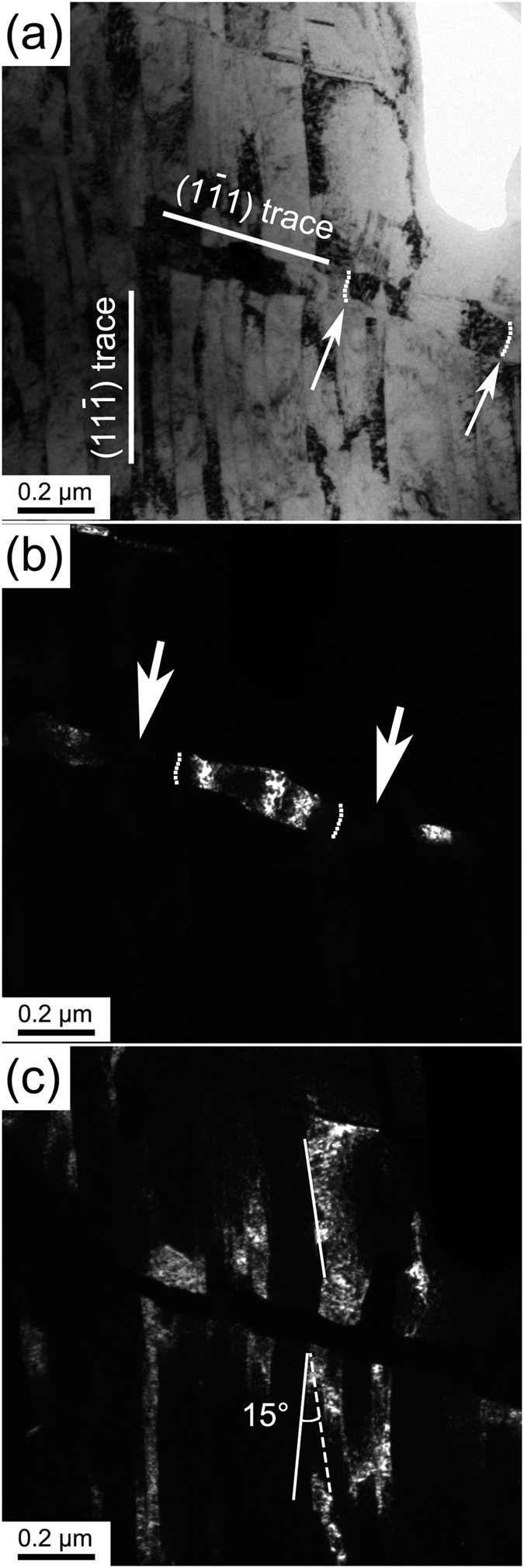
(**a**) A bright-field TEM image of a group of NBs passing through a single NB. The two solid lines mark two crystallographic planes of the matrix. The dashed lines marked by white arrows indicate longitudinal dislocation walls; (**b**) the corresponding dark-field image of the single NB. The fragments of the single NB show a tendency to be constricted at the location of the longitudinal boundaries (marked by the arrows). The dashed lines indicate longitudinal dislocation walls; and (**c**) the corresponding dark-field image of the group NBs. Two solid tangent lines of a NB were draw in its upper and lower parts, respectively, and one dashed black line was also drawn parallel to tangent line of the upper part to measure the distortion angle of the NB.

With increasing strain, the misorientation between NBs and the matrix increased, and some NBs were divided into several segments due to NBs interactions. Figure 4a shows a group of NBs passing through a single NB with a high density of dislocations. The single NB was cut into several parts (shown by the dark-field image in Fig. 4b). Both of the single NB and the group of the NBs (shown by the dark-field image in Fig. 4c) are aligned roughly along a {111}. It can be seen from Fig. 4b that the single NB is divided into several fragments with different grain orientations, and the fragments show a tendency to be constricted at the location of the longitudinal boundaries (two clear examples are indicated by the arrows in Fig. 4b). This could result from the fact that the dislocations accumulated at the interacting boundaries to form longitudinal dislocation walls in the NB as “bamboo nodes”. Two examples of the “bamboo nodes” are marked with dashed line and indicated by the arrows in the bright-field image in Fig. 4a and dashed lines in corresponding dark-field image in Fig. 4b. These “bamboo-node” dislocation walls would evolve into large-angle boundaries, leading to the segmentation of the NBs with increasing strain^35^. These segments would gradually converted to equiaxed grains or subgrains through dislocation slip and sub-grain rotations. The dark-field image in Fig. 4c shows that the group of NBs was no longer straight but severely distorted. A typical individual NB in the group of the NBs had a distortion angle of about ~15°, which is indicated in Fig. 4c by drawing two tangent lines of the upper part and lower part of the NB and then measuring the angle between them. This is due to the increased dislocation generation and annihilation in the boundaries of the NBs with increasing strain, thereby to increase the misorientation angles between the NBs.

**Figure 5:**
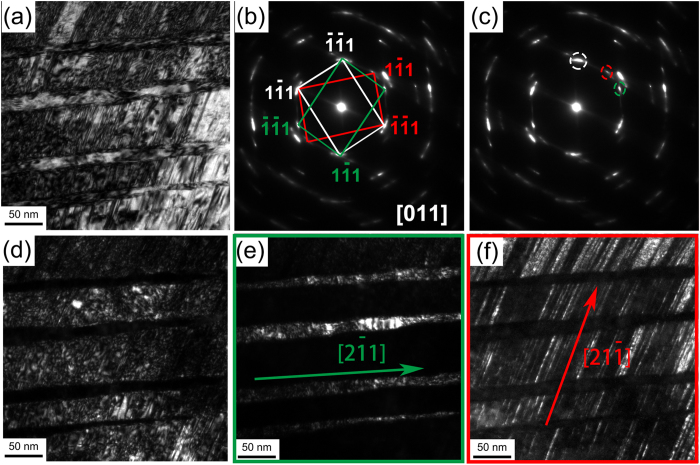
(**a**) Secondary SFs/nanotwins inclined to the transversely aligned primary twins with a high density of dislocations; (**b**) a corresponding [011] SAED pattern showing three sets of reciprocal lattices from the matrix and the twins; (**c**) three diffraction spots marked with white, green and red dotted circles were chosen for dark-field imaging shown in (**d**) the matrix (white frame), (**e**) the primary twins (green frame) and (**f**) the secondary SFs/nanotwins (red frame), respectively.

Figure 5a shows clear evidence of the formation of a high density of SFs/nanotwins inclined to the transversely aligned TBs. A high density of dislocations was also identified. For the convenience of the discussion in this paper, the twins formed in stages 1 and 2 are all denoted as primary twins, whereas the thin SFs/nanotwins within and inclined to the primary twins are denoted as secondary SFs/nanotwins. The [011] SAED pattern in Fig. 5b confirms the coexistence of the two sets of twins comprising primary twins and secondary SFs/nanotwins in Fig. 5a. Three diffraction spots for dark-field imaging for Fig. 5d,e and f are marked by white, green and red dashed circles in Fig. 5c, respectively. Based on the crystallography of FCC structures, the orientations or twinning direction of the primary twins and the secondary SFs/nanotwins are 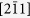 (green color, Fig. 5e) and 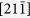 (red color, Fig. 5f), respectively. The SAED pattern in Fig. 5c shows that the matrix has the strongest spots, and the spots of the matrix and the primary twins appeared as short arcs, indicating small-angle misorientation in both the matrix and the twins. No secondary SFs/nanotwins was found within the narrow primary twin/matrix lamellae with width less than ~7 nm. It can be seen from Fig. 5f that the secondary SFs/nanotwins were extremely thin, no more than a few nanometers in thickness, which was probably caused by the size effect of the twinned volume because a much higher stress may apply within the nanoscale matrix between primary twins^36^.

**Figure 6:**
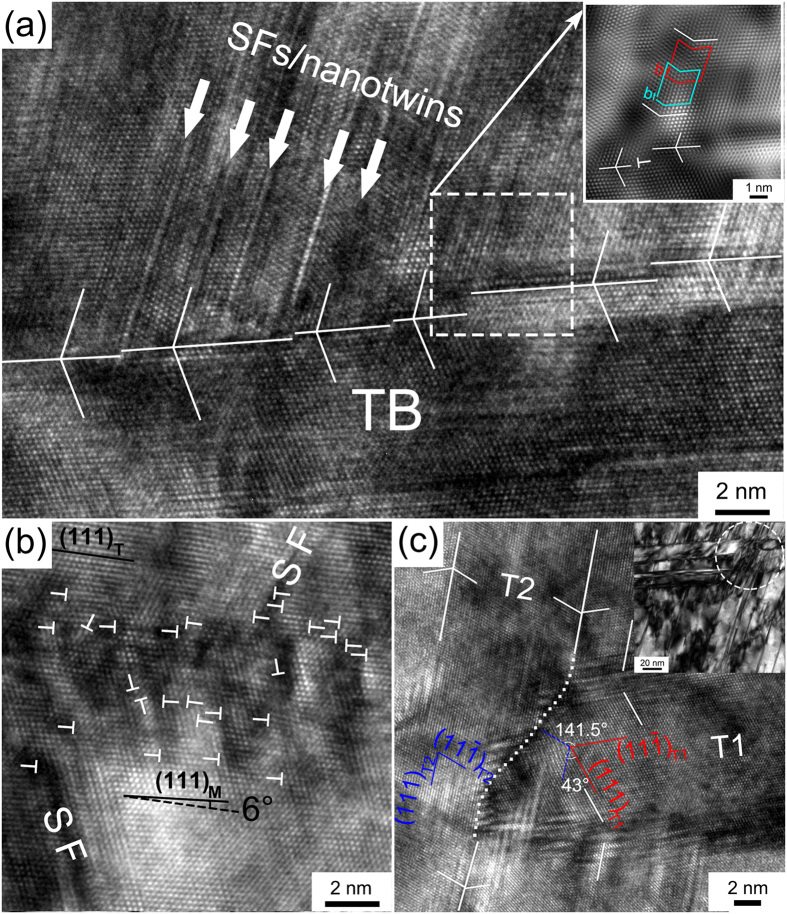
(**a**) An HRTEM image showing a high density of inclined SFs/nanotwins interacting with a primary TB. White arrows mark some of the inclined SFs/nanotwins. The inset is a Fourier filtered image of the area enclosed by the square, showing the presence of Shockley partials (Burgers circles are drawn by blue and red lines) at the secondary TB and a dislocation (marked by white “T”) near the primary TB. TBs and twin relationships are indicated by white lines; (**b**) an HRTEM of a primary TB showing a high density of dislocations (indicated by “T”) accumulated at the TB. Two black solid lines were drawn parallel to {111} at each side of the TB, respectively, and one dash black line was also drawn parallel to (111)_T_ to show the misorientation between the {111} planes across the TB; (**c**) a typical HRTEM image presents that a secondary twin (T2) traverses a primary twin (T1), showing a misorientation between the {111} planes of both T1 and T2 (indicated by red and blue lines, respectively) near the interacted TB (dashed white line). TBs and twin relationships are indicated by white solid lines. Inset is a bright-field image of secondary twins traversing the primary twins (marked by a dashed circle).

The HRTEM image (Fig. 6a) shows the reactions between the inclined secondary SFs/nanotwins and primary twins. The atomic arrangement at primary TBs was distorted by dislocation–TB interactions at the intersection area of the secondary SFs/nanotwins and primary twins. The occurrence of dislocation–TB interactions at the intersection of the secondary SFs/nanotwins produced Shockley partial dislocations on several consecutive {111} atomic layers parallel to primary TB, leading to steps along the primary TB and de-twinning of the primary twin^37^. The Fourier filtered image in inset of Fig. 6a obtained from the outlined area shows that several consecutive atoms on {111} plane were slightly distorted. The Burgers circuit indicates that partial dislocations of SFs exist next to a dislocation (marked by a “T”). Thus, various reactions might have occurred when SFs/nanotwins were driven towards the primary TB^38^,^39^.

After dislocation–TB interactions, a very high density of dislocations accumulated at and near primary TBs, as shown in Fig. 6b. With the accumulation of dislocations at the TB, the originally parallel {111} planes at the two sides of the TB were no longer parallel to each other, but had a deviation angle of ~ 6°. The 6° deviation angle is indicated in Fig. 6b by drawing two black lines parallel to the (111) planes of the twin (marked as (111)_T_) and the matrix (marked as (111)_M_). The originally coherent TBs lost their coherency and transformed into incoherent high-angle GBs after accumulating a large number of dislocations at and near TBs^11^,^40^. Cao *et al*.^41^ proposed a new GB formation mechanism that the remnant dislocations from dislocation–TB reactions after the fully de-twinning of primary twins can generate dislocation walls that form low-angle GBs.

In addition, some of these secondary nanotwins traversed the primary twins, as shown in the dashed circle area of a bright-field image in upper right inset of Fig. 6c. A detailed HRTEM image is given in Fig. 6c. It is seen from Fig. 6c that a secondary nanotwin (marked as T2) with a thickness of ~10 nm traversed the primary twin (marked as T1). After interaction between T1 and T2, the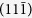 plane (marked as 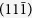
_*T*2_) of T2 interaction area still form a twin relationship with the 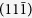 plane (marked as 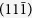
_*T*1_) of T1, whereas the (111) plane of T2 and T1 has a misorientation about 43°. It can thus be concluded that with further deformation, secondary nanotwins could divide primary twins into many equiaxed parts and transform the primary equiaxed parts into an equiaxed NC structure, and the distorted secondary TB (marked as white dashed line in Fig. 6c) in the interaction area would evolve into high-angle GBs.

### Deformation stage 4 (ε = ~4.0–6.5): the equilibrium microstructure

**Figure 7:**
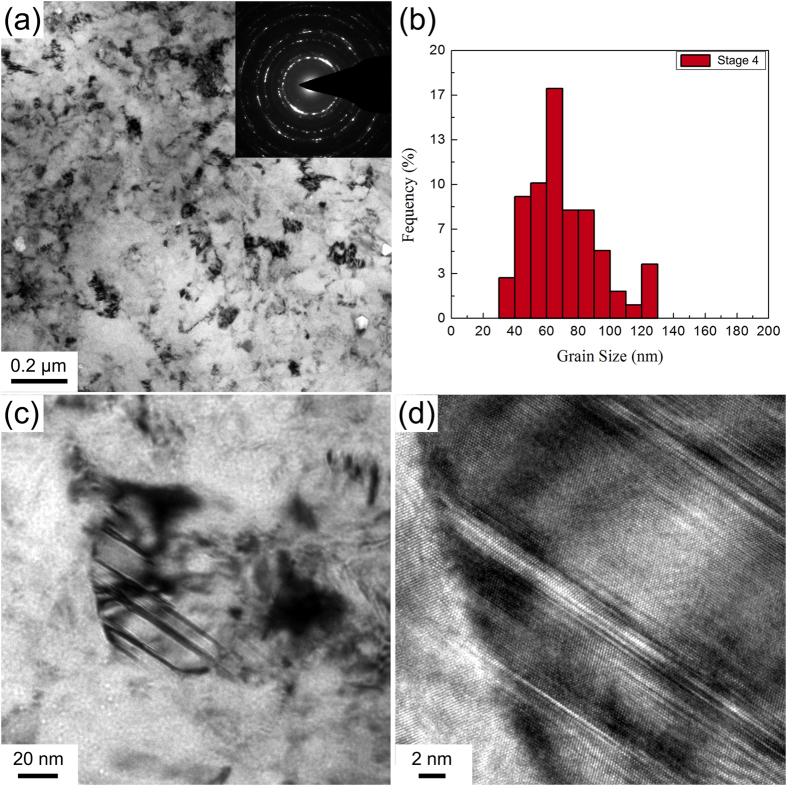
(**a**) A typical TEM image and corresponding SAED pattern in deformation stage 4; (**b**) the statistical grain size distribution; (**c**) a typical NC grain containing SFs and nanotwins; and (**d**) an HRTEM image of the NC grain in (**c**).

At deformation stage 4, the microstructure reached an equilibrium state. Figure 7a shows the equilibrium homogeneous equiaxed NC structure. The corresponding ring-like SAED pattern in the inset in Fig. 7a indicates that the structure contains small grains with random grain orientations. Figure 7b presents the grain size distribution of the alloy at this deformation stage. The average grain size is ~68 nm with the sizes ranging from ~30 to 120 nm. Figure 7c shows a typical equiaxed NC structure with a grain size of ~90 nm. A high density of SFs and nanotwins with a few atomic layers existed in the equiaxed NC grains, as shown in Fig. 7d.

## Discussion

Based on the above-mentioned deformation stages, it is feasible to propose a mechanism for grain refinement in the FCC FeCoCrNi HEA with a low SFE processed by HPT. The main microstructural evolution mechanisms are concurrent NB subdivision and deformation twinning that result in significant grain refinement from an average grain size of ~25 μm in the as-received sample to ~68 nm at deformation stage 4, leading to impressive ~350 times reduction in the grain size.

Our experimental results indicated that the alloy deformed predominantly by dislocation slip and twinning. Dislocation slip can be wavy or planar depending on the SFE of materials. For materials with high SFEs, such as Al^42^ and Ni^43^, wavy slip occurs that leads to the formation of dislocation cells, cell blocks and dense dislocation walls. For materials with low SFEs, e.g., the HEA investigated here^21^, because all full dislocations dissociate into partials with a wide stacking fault between each pair of dissociated partials, cross-slip becomes very difficult and therefore slip is planar. In planar slip, dislocations rearrange themselves and form a finely organized Taylor lattice structure at low strains^44^. With increasing strain, the Taylor lattice structure evolves into Taylor lattice domain, with the domain boundary being a single dislocation wall consisting of geometrically necessary dislocations to accommodate the misorientation between the Taylor lattice domains^44^. A second dislocation wall parallel the first dislocation wall forms with further increasing strain in order to shield the stress relating to the domain boundaries, and the region between the double walls is eventually produced and called a microband^31^,^45^.

Planar slip is promoted by short range ordering (SRO), low SFE and large lattice friction stress^17^. The equiatomic FeCoCrNi HEA has a low SFE^21^ and large lattice friction stress^46^ bot no SRO in the lattices^27^. Therefore, the presence of planar slip and NBs in the FeCoCrNi HEA is attributed to the combined effect of low SFE and large lattice friction stress.

It’s worth noting that NBs were observed in the initial deformation stages with extremely low strain (Fig. 1a), while scarcely any Taylor lattice structure or domain boundary was observed during HPT in this work. The intrinsic lattice distortion in the HEA, which is caused by the atomic size variation of the constituent elements and the variation in binding energy between atom pairs, act as obstacles to dislocation motion^47^. During deformation, dislocation lines in HEAs with varying energy state (caused by the introduction of dislocation in the already strained lattice) have to move through the distorted lattice with fluctuating energy states, hence low energy dislocations with large energy barriers will easily become immobile and act as pinning points^47^. Also, Edalati *et al*.^48^ reported that solute atoms can increase the local stress needed for dislocation motion, and thus reduce dislocation recovery, recrystallization and grain boundary migration, and promote dislocation accumulation. Thus, the introduction of dislocations in the already strained FeCoCrNi solid solution lattice readily promotes dislocation accumulation and the subsequent formation of dislocation walls, leading to the formation of NBs. This explains the observed small width of NBs (~65 nm), compared to that of microbands in conventional alloys, such as rolled Al-Mg alloy^31^ and cyclic extrusion compressed AlCu4Zr^49^.

Because of the low SFE of the HEA, twinning is an important deformation mode in the coarse-grained HEA. Therefore, deformation twins with thicknesses of only a few tens nanometers were frequently observed at the very early stages of the deformation process. Twinning at these early stages of deformation should occur via a mechanism that operates in coarse-grained materials^50^. Twin–twin interactions were frequently observed in FCC materials with low SFEs, including stainless steels^51^ and Co^52^. Significant twin–twin interactions together with dislocation–TB interactions lead to significant grain refinement.

With increasing strain, abundant thin secondary SFs/nanotwins inclined to the primary TBs formed (Fig. 5a). Activation of another twinning system other than the primary twinning system is possible when the crystalline orientation relative to the shear stress direction is favorable^36^. Another reason is that the twinning mechanism in nanocrystalline materials is different from that in coarse-grained materials and the critical resolved shear stress for twinning could be smaller than that of full dislocation activities in nanocrystalline materials^20^. Note that no secondary SFs/nanotwins is found within narrow primary twin/matrix lamellae with width smaller than ~7 nm (Fig. 5f), which is similar to the result reported in ref. 41. In NC FCC materials, deformation twinning occurs mostly via partial dislocation emissions from GBs^20^. An inverse grain size effect on twinning was reported that twinning becomes more difficult in grains smaller than a critical size since higher stress is required to emit partials from GBs of grains below a certain critical grain size^53^. In Fig. 5, secondary SFs/nanotwins formed via the emissions of partial dislocations from primary TBs. However, when the primary twin/matrix lamellae is extremely thin (small than ~7 nm in Fig. 5e), emissions of partials from primary TBs becomes difficult and thus no secondary SF/nanotwin was observed within narrow primary twin/matrix lamellae.

The formation mechanism of steps along TBs (Fig. 6a) during dislocation–TB interaction has been studied previously^54^,^55^. Numerous experiments^40^ and molecular dynamics simulations^56^,^57^ showed that when an extended dislocation is forced by an external stress into a coherent TB, it recombines or constricts into a perfect dislocation configuration at the coherent TB and then slips through the boundary by splitting into three partials. Two of them glide in the slip plane of the adjacent twin lamella, constituting a new extended dislocation, while the third one (the twinning partial) glides along the TB to form a step^40^. Cao *et al*.^41^ reported that the occurrence of a dislocation–TB interaction at the intersection of the secondary and primary twins produced a Shockley partial dislocation moving along the primary TB, leading to de-twinning by one atomic layer of the primary twin. As a result of de-twinning, the primary twins width in Fig. 5e from left to right varied.

During the HPT process, the hardness increased continuously with increasing the equivalent strain before reaching a plateau (see in Supplementary Fig. S1), indicating a strain hardening process in deformation stages 2 and 3. Both dislocation activities and deformation twinning contribute to the strain hardening process. It is proposed previously that the formation of NBs in deformation stages 2 and 3 can inhibit dislocation motion and lead to high strain hardening^44^. Dislocation accumulation at twin–twin interacting region and the formation of L-C locks in deformation stage 2 derive their effectiveness in the strain hardening^58^. In deformation stage 3, secondary SFs/nanotwins were constrained by primary twins, and the latter served as effective barriers for the secondary twinning process, which contributed to the strain hardening process^59^. Meanwhile, dislocation–TB interactions increased the density of dislocations in grains, which should also have a positive effect on the strain hardening process.

**Figure 8:**
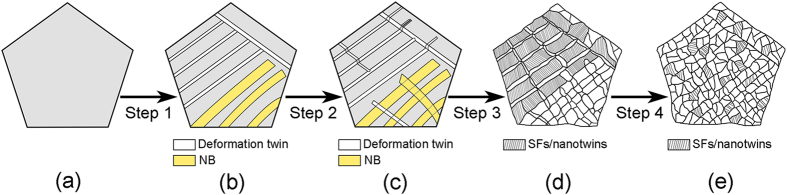
A schematic diagram of the HPT-induced grain refinement process.

The microstructural evolution of FeCoCrNi HEA is divided into four deformation stages. The grain refinement process can also be described in four steps, as illustrated schematically in Fig. 8. The as-received alloy was of a coarse-grained structure, as shown in Fig. 8a. During grain refinement step 1, grains retained roughly their original sizes. NBs and primary deformation twins formed in the coarse grains immediately after applying a high pressure of 4 GPa for 1 minute without torsional treatment. One or two twinning systems were activated in each grain, depending on the orientation of individual grains that affects the resolved shear stresses on different twinning systems. NBs and twins are presented as yellow and white ribbons, respectively, in Fig. 8b. During grain refinement step 2, three-types of the interactions occurred, including NB–NB, twin–NB and twin–twin interactions, as illustrated in Fig. 8c. NB–NB interactions resulted in distortion of NB boundaries. The twin–NB interaction created the subdivision or re-launching of the twins. Twin–twin interactions resulted in severe lattice distortion and plenty of dislocations accumulated in the interaction areas. During grain refinement step 3, NBs were divided into several segments (Fig. 8d) due to the NBs interaction, and finally the elongated segments were transformed into equiaxed subgrains or grains through dislocation slip and sub-grain rotation. Additionally, abundant extremely thin secondary SFs/nanotwins inclined to the primary TBs were formed, as illustrated schematically in Fig. 8d. The remaining primary TBs transformed into incoherent high-angle GBs through dislocation–TB interactions and some secondary nanotwins divided the primary twins into many equiaxed parts. During grain refinement step 4, a homogeneous equiaxed NC structure was obtained, as illustrated in Fig. 8e. SFs/nanotwins in nanocrystalline grains were continuously formed through partial dislocation emission from the GBs.

## Conclusions

The microhardness and microstructural evolution of an FeCoCrNi HEA processed by HPT was investigated in detail. The following conclusions are drawn:

1. Different from the SPD-induced grain refinement mechanisms in conventional materials, the SPD-induced grain refinement mechanism in the HEA includes concurrent NB subdivision and deformation twinning. The concurrent deformation mechanism causes significant grain refinement from an average grain size of ~25 μm in the as-received sample to ~68 nm.
2. The misorientation between a NB and its neighboring matrix increases with increasing strain because of dislocation accumulation at the boundaries of the NB with increasing strain. The interaction between two sets of NBs on different slip planes causes boundary distortion and segmentation of NBs.
3. The interaction between the tips of secondary twins and the boundaries of primary twins leads to the emission of new secondary twins on the other side of the primary twins to release local stress concentration. High densities of dislocations accumulated in the interaction areas result in local heavy lattice distortion.
4. Significant dislocation–TB reactions produce atomic steps at primary twin boundaries that gradually transform the originally coherent TBs into incoherent high-angle GBs.

## Methods

### Sample preparation

The FeCoCrNi HEA used in this investigation was fabricated by a powder metallurgy route. The mixed high purity Fe, Co, Cr and Ni raw materials with an equiatomic ratio were induction melted in vacuum, and then, the melt were disintegrated by high pressure argon into fine liquid particles. The droplets were then cooled down in a stainless steel chamber. The pre-alloyed FeCoCrNi powder was collected and sieved, and filled in a steel can. The canned powder was hot extruded at 1200 °C with an extrusion ratio of 6:1 to obtain a billet with a high density. Details of the fabrication technique and the characterizations of the powder have been reported in our previous study^60^. The actual composition of the FeCoCrNi billet is very close to the nominal composition (see in Supplementary Table S1). The consolidated alloy has a fully recrystallized microstructure, with an average grain size of ~25 μm and a large number of the annealing twins (see in Supplementary Fig. S2).

The as-received alloy was firstly cut into several disks along the transverse direction with a diameter of 10 mm and thickness of about 1.2 mm. The disks were ground carefully on each side with 1000 grit and 2000 grit SiC paper sequentially, resulting in a smooth surface and a final specimen thickness of ~1 mm for HPT processing. An unconstrained HPT die set was used in our experiments. The disks were subjected to pressure along the normal direction in a non-hydrostatic environment^61^. Slippage was not found during the HPT processing. Four specimens were compressed under 4 GPa with N = 0, 1, 2, 4 revolutions. And among them N = 0 indicates that the sample was only applied with a pressure of 4 GPa for ~1 minute without any torsional treatment. Due to the material outflow during the HPT processes, the final thicknesses of the disks were approximately 0.95 mm, 0.68 mm, 0.58 mm, and 0.47 mm, respectively.

### Characterization techniques

The microhardness measurements were taken along the radii from the center to edge at different radial directions (distance between two adjacent indentations is 0.5 mm) using a load of 100 g for 10 s. The structures of both the as-received and the deformed samples were characterized using an X-ray diffractometer (XRD, Dmax 2500 VB) with Cu-Kα radiation and a TEM (Tecnai G2 F20). XRD results showed that both the as-received and deformed samples have an FCC single phase. Samples for TEM observations were cut along a radius direction of the disks. The location of each sample center relative to its disk center was recorded in order to calculate the HPT strain. These samples were thinned to a thickness of ~70 μm, and then electropolished using a solution of 5% perchloric acid and 95% alcohol at −40 °C with an applied voltage of 27 V. Grain sizes were measured directly from the TEM images by measuring the width of the individual grains along linear traverses. The average grain sizes of samples after deformation were estimated from at least 200 grains. For each deformation stage, the average twin/NB thicknesses were estimated from up to 20 TEM and HRTEM images.

## Additional Information

**How to cite this article:** Wu, W. *et al*. Dual mechanisms of grain refinement in a FeCoCrNi high-entropy alloy processed by high-pressure torsion. *Sci. Rep.* **7**, 46720; doi: 10.1038/srep46720 (2017).

**Publisher's note:** Springer Nature remains neutral with regard to jurisdictional claims in published maps and institutional affiliations.

## Supplementary Material

Supplementary Information
