# Supplementary material for: Dual mechanisms of grain refinement in a FeCoCrNi high-entropy alloy processed by high-pressure torsion
Source: Sci Rep. 2017 Apr 21;7:46720. doi: 10.1038/srep46720 (PMC5399454; doi:10.1038/srep46720)
Supplement: Supplementary Information [file srep46720-s1.pdf]

Supplementary Information

Dual mechanisms of grain refinement in a FeCoCrNi high-entropy alloy processed by high-pressure torsion

Wenqian Wu [1], Min Song [1,*], Song Ni [1], Jingshi Wang [1], Yong Liu [1,*], Bin Liu [1], Xiaozhou Liao [2]

[1] State Key Laboratory of Powder Metallurgy, Central South University, Changsha 410083, China

[2] School of Aerospace, Mechanical and Mechatronic Engineering, The University of Sydney, Sydney, NSW 2006, Australia

*Corresponding authors.

Email addresses: msong@csu.edu.cn (M. Song), yonliu@csu.edu.cn (Y. Liu)

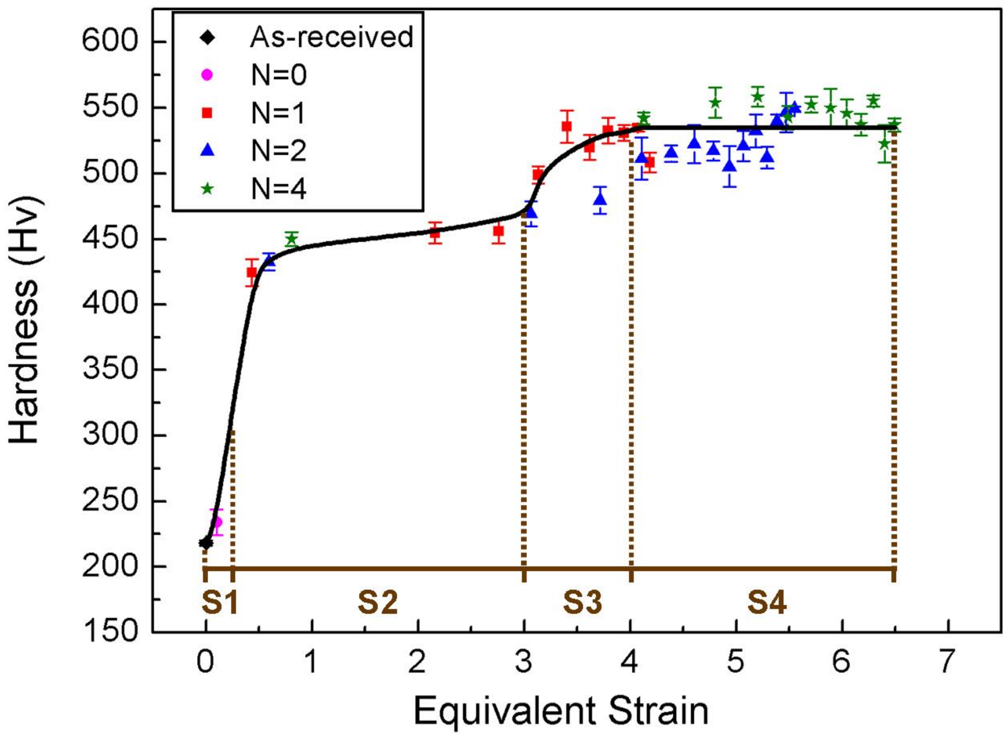

Supplementary Figure 1: Relationship between microhardness and the equivalent strain.

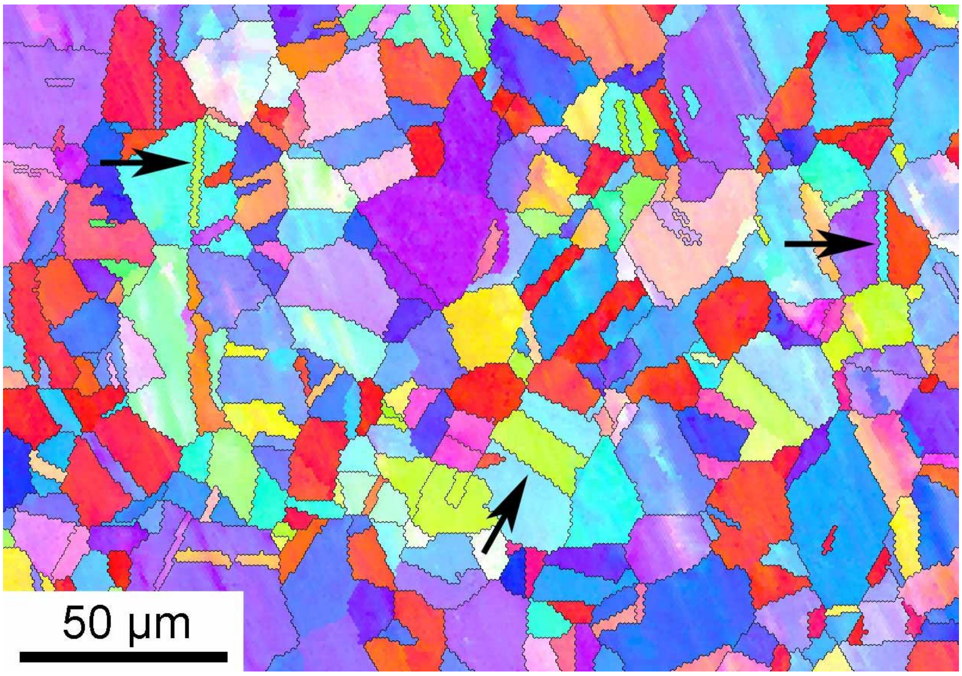

Supplementary Figure 2: An EBSD map of the as-received sample. The consolidated alloy has a fully recrystallized microstructure, with an average grain size of ~25 μm and a large number of the annealing twins. The twins can be easily identified by both the grain morphology – having parallel twin boundaries – and EBSD patterns relative to that of the nearby matrix. Some twins are indicated by arrows.

Supplementary Table 1

Chemical compositions of the FeCoCrNi billet

Elements	Fe	Co	Cr	Ni
at.%	24.5	24.5	24.3	26.7
